# Non-O1, Non-O139 *Vibrio cholerae* Bacteremic Skin Infection with Multiple Skin Necrosis: Case Report

**DOI:** 10.3390/tropicalmed10040110

**Published:** 2025-04-17

**Authors:** Amer Ibrahim Alomar, Nasreldin Elhadi, Lamya Zohair Yamani, Reema Allahham, Rana Alghamdi, Ibrahim Alhabib, Asim Diab, Nehal Mahmoud, Bashayer AlDossary, Mariam Almejhim, Nouf Al-Romihi, Faye Aldehalan, Reem Al Jindan

**Affiliations:** 1 Department of Clinical Laboratory Science, College of Applied Medical Sciences, Imam Abdulrahman Bin Faisal University, Dammam 31441, Saudi Arabia; aiomar@iau.edu.sa (A.I.A.); lzyamani@iau.edu.sa (L.Z.Y.); reemalahham97@icloud.com (R.A.); ranzilla35@gmail.com (R.A.); ikalhabeeb@iau.edu.sa (I.A.); faaldehalan@iau.edu.sa (F.A.); 2 Department of Microbiology, College of Medicine, Imam Abdulrahman Bin Faisal University, Dammam 31441, Saudi Arabia; asimdiab@hotmail.com (A.D.); nmhosin@iau.edu.sa (N.M.); raljindan@iau.edu.sa (R.A.J.); 3 Diagnostic Microbiology Laboratory, King Fahd Hospital of the University, Imam Abdulrahman Bin Faisal University, Al Khobar 34445, Saudi Arabia; bhdossary@iau.edu.sa (B.A.); mimejhim@iau.edu.sa (M.A.); nromihi@iau.edu.sa (N.A.-R.)

**Keywords:** *V. cholerae*, case report, cholera toxin, wound infection, skin necrosis

## Abstract

Non-O1, non-O139 *Vibrio cholerae* (NOVC) extraintestinal infections are rare, but recently, several clinical incidents have been reported worldwide. Toxigenic *V. cholerae* is a well-known etiological agent of cholera, responsible for acute dehydrating watery diarrhea. Outbreaks occur in an epidemic seasonal pattern, particularly in countries with poverty and poor sanitation. Strains of NOVC are usually not involved in causing the epidemic or pandemic outbreaks seen with potential strains of *V. cholerae* serogroup O1 and O139. However, they can still cause severe sporadic cases of intestinal as well as extraintestinal infections. In this study, we investigated a case of extraintestinal infections associated with the NOVC serogroup isolated from a deep closed wound abscess. The isolate was screened for the presence of three major virulence genes, *tox*R, *ctx*A, and *tcp*A. The strain tested positive for the *tox*R gene encoding the regulatory protein and cholera toxin (*ctx*) gene and tested negative for the toxin-coregulated pilus (TCP) gene, which is essential for the colonization of the human intestine, causing the severe diarrheal disease cholera. To the best of our knowledge, this is the first case of extraintestinal infection caused by toxigenic *Vibrio cholerae* non-O1/non-O139 in a hospitalized patient in Saudi Arabia.

## 1. Introduction

*Vibrio cholerae* is Gram-negative bacteria belonging to the family *Vibrionaceae*, which is naturally distributed in aquatic environments. To date, there are more than 200 serogroups based on the structure of the O antigen and only serogroups O1 and O139 are responsible for causing fatal diarrheal disease in humans worldwide [1]. Strains of *V. cholerae* are capable of secreting cholera toxin (*ctx*), responsible for more than 4 million cases with about 20,000 to 150,000 deaths per year [2,3]. *V. cholerae* strains that do not agglutinate with O1 and O139 antiserum are collectively classified as non-O1 and non-O139 *V. cholerae* (NOVC) [3,4].

NOVC serogroups are not as closely monitored by health authorities as O1 and O139 *V. cholerae* due to their tendency to cause isolated cases or localized outbreaks with less severe and often self-limiting symptoms. These strains are missing one or both of the main virulence factors, cholera toxin (CT) and toxin-coregulated pilus (TCP), in their genomes [5,6,7,8]. However, NOVCs are becoming increasingly significant in the field of public health on a global scale. Various studies have indicated a rise in the number of cholera-like diarrhea and extraintestinal infections such as bacteremia, otitis media, wound and soft tissue infections, and outbreaks caused by NOVC, which is linked to the gradual increase in seawater temperatures [9,10,11,12,13,14,15].

NOVC bacteremic skin infection accompanied by skin necrosis is rare; however, several reports of extraintestinal infections associated with these serogroups have been reported recently. Invasive, life-threatening NOVC bacteraemia primarily occurs in high-risk individuals, including immunosuppressed patients and those with advanced lung cancer and underlying liver disease [16,17,18,19,20]. Human activity in coastal areas contributes to this trend, as does the growing international trade of seafood [5], the popularity of consuming raw seafood, and the growing population of immunocompromised individuals, such as elderly people with pre-existing conditions, in particular [21]. In this study, we report a case of a bacteremic skin infection with multiple skin necrosis caused by a toxigenic strain of NOVC in a hospitalized patient.

## 2. Case Presentations

The patient is a 66-year-old female with a medical history of type II diabetes, hypothyroidism, and end-stage renal disease (ESRD) and is currently on hemodialysis. She has hypertension and has been newly diagnosed with heart failure (ejection fraction (EF) 36%). The patient suffered from a stroke 5 years ago with residual weakness in her left lower limb and was also diagnosed with COVID-19 pneumonia. She was on warfarin, but the doctor advised her to discontinue taking it, as it may have been the cause, and was started on clexane (enoxaparin sodium). Three months prior to admission, she complained of skin redness, then ulceration, then necrosis, which extended from her legs to the abdomen and breast. She also presented to the emergency room (ER) on 25 October 2020 with fever and multiple skin necrosis. She was admitted and noted as having multiple skin necrosis to rule out calciphylaxis with superimposed bacterial infection. Computed tomography (CT) abdomen was performed, in which no intraabdominal issues were observed. The infectious diseases team was consulted, and they started her empirically on meropenem and clindamycin. Laboratory investigation tests revealed leukocytosis, with a white blood cell count of 17.8 k/μL, and an elevation of C-reactive protein (26.71 mg/dL) and Procalcitonin (4.46 μg/mL), as presented in Table 1.

**Figure 1:**
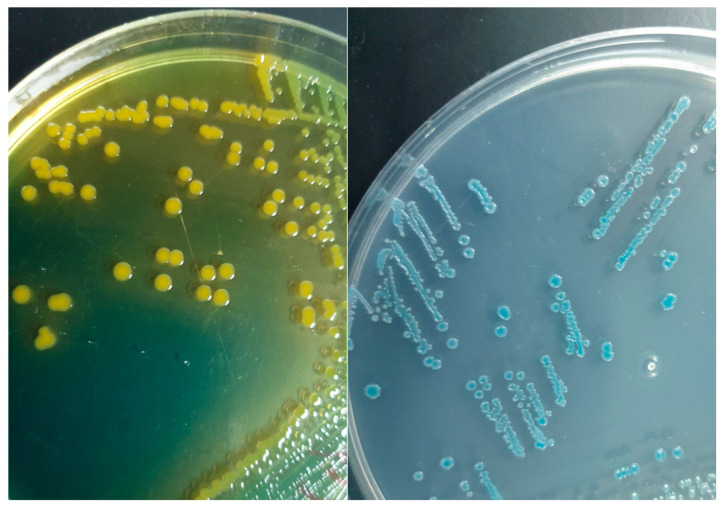
**Left**: Appearance of yellow colonies of *V. cholerae* non-O1/O139 strain isolated from wound infection on thiosulfate–citrate–bile salts–sucrose (TCBS) agar. **Right**: Appearance of green-blue to turquoise-blue colonies of *V. cholerae* on CHROMagar Vibrio.

The initial wound culture was taken on 29 October 2020 and sent to the microbiology laboratory. The microscopic examination revealed +1 epithelial cells and +1 WBCs and the Gram stain showed Gram-negative bacilli. According to the internal policies and procedures of the microbiology laboratory at King Fahd Hospital of the University (KFHU), the wound swab specimen was sub-cultured on sheep blood, MacConkey, and anaerobic Brucella agars; after 24 h, growth on all plates was identified by matrix-assisted laser desorption/ionization time-of-flight mass spectrometry (MALDI-TOF-MS) (VITEK MS; bioMérieux) and the Knowledge Base database (version 3.0) with a confidence value of 99.9% to identify *V. cholerae*. Meanwhile, the organism was grown on thiosulfate–citrate–bile salts–sucrose (TCBS) agar and Vibrio chromogenic agar, showing yellow colonies on the TCBS agar and green-blue to turquoise-blue on the Vibrio chromogenic agar (Figure 1).

**Figure 2:**
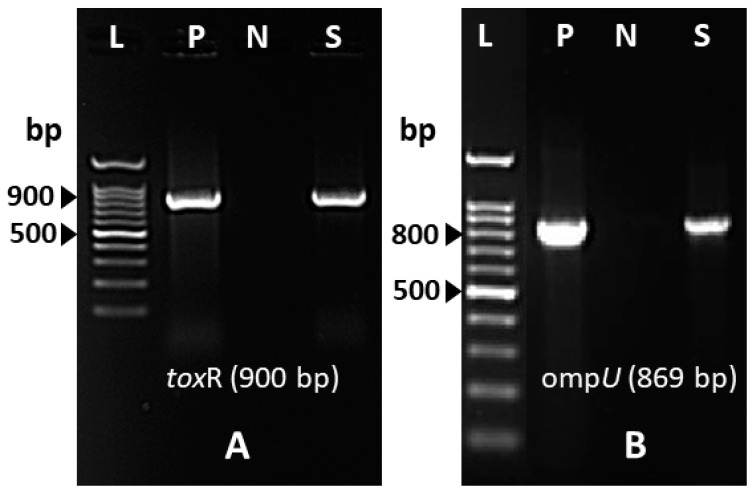
(**A**) PCR assay detected the *tox*R gene of *V. cholerae* non-O1/O139 strain isolated from wound infection. Lane L, 100 base pair (bp) DNA ladder; lane P, *V. cholerae* O1 Ogawa-Classical NIH41 (positive control for *tox*R gene); lane N, negative control; lane S, *V. cholerae* non-O1/O139 strain isolated from wound infection. (**B**) PCR detected the *omp*U gene of *V. cholerae* non-O1/O139 strain isolated from wound infection. Lane L, 100 base pair (bp) DNA ladder; lane P, *V. cholerae* O1 Ogawa-Classical NIH41 (positive control for *omp*U gene); lane N, negative control; lane S, *V. cholerae* non-O1/O139 strain isolated from wound infection.

**Figure 3:**
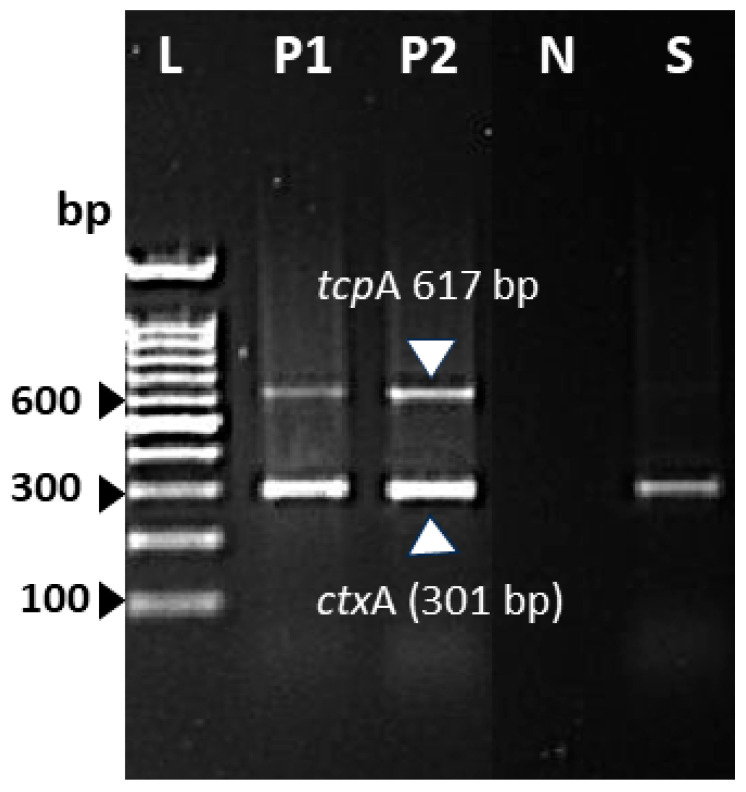
Multiple PCR assay detected the *ctx*A and *tcp*A genes of *V. cholerae* non-O1/O139 strain isolated from wound infection. Lane L, 100 bp DNA ladder; lane P1, *V. cholerae* O1 Ogawa-Classical NIH41 (positive control for *ctx*A and *tcp*A gene); P2, *V. cholerae* O1 Inaba-Classical NIH35A3; lane N, negative control; lane S, *V. cholerae* non-O1/O139 strain isolated from wound infection.

**Figure 4:**
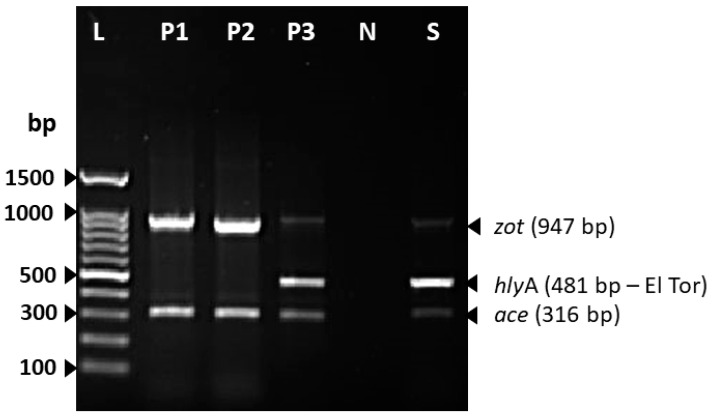
Multiplex PCR assay detected the *zot*, *ace*, and *hly*A genes of *V. cholerae* non-O1/O139 strain isolated from wound infection. Lane L, 100 bp DNA ladder; lane P1 and P2, *V. cholerae* O1 Ogawa-Classical NIH41 and *V. cholerae* O1 Inaba-Classical NIH35A3 (positive control for *ace* and *zot* genes); lane P3, *V. cholerae* O139 MO45 (positive control for *ace*, *hly*A, and *zot* genes); lane N, negative control; lane S, *V. cholerae* non-O1/O139 strain isolated from wound infection.

**Figure 5:**
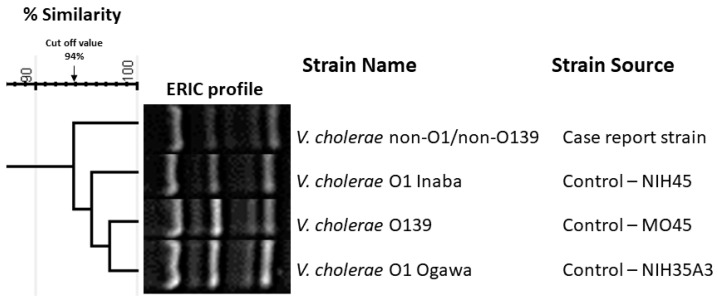
Cluster of ERIC-PCR fingerprints of *V. cholerae* non-O1/O139 strain isolated from wound infection and control strains of *V. cholerae* O1 Ogawa-Classical NIH41, *V. cholerae* O1 Inaba-Classical NIH35A3, and *V. cholerae* O139 MO45. The genetic similarities between the fingerprints generated by ERIC primer were calculated using the cosine coefficient, and the fingerprints were grouped according to their similarities using the unweighted pair group method with arithmetic means algorithm (UPGMA). The scale bar at the top of the dendrogram shows the cosine coefficient of genetic similarity (%) and the arrow above the percentage similarity scale indicates the cutoff value of 94% for cluster analysis. The toxigenic strain *V. cholerae* non-O1/O139 isolated from the wound infection showed 94% genetic similarity with the three control strains of *V. cholerae* O1 Ogawa, *V. cholerae* O1 Inaba, and *V. cholerae* O139.

The isolated strain was serotyped using antiserum (MAST, ASSURE Antiserum, Liverpool, UK) for serotyping *V. cholerae* O1 and O139. The isolated strain slide agglutination test with polyvalent antiserum was negative and reported as being the non-O1/non-O139 serotype. Antimicrobial susceptibility testing was conducted with the Vitek2 system (Biomèrieux, France) and the strain was found to be susceptible to ampicillin, ciprofloxacin, and trimethoprim-sulfamethoxazole. The isolated strain was further investigated using polymerase chain reaction (PCR) for virulence gene determinants including toxin regulon (*tox*R), outer membrane protein (*omp*U), cholera toxin (*ctx*), toxin-coregulated pilus (*tcp*A), accessory colonization enterotoxin (*ace*), hemolysin (*hly*A), and zonula occludens (*zot*). The primers used in this study and the expected amplicon sizes are listed in Table 2. The isolated strain was reported to be positive for *tox*R and *omp*U (Figure 2A,B), whereas Figure 3 shows the presence of *ctx* genes while the genes for *tcp*A are absent. Among other virulence genes, the isolated strain carried the *ace*, *hly*A, and *zot* genes, as presented in Figure 4. To determine the genetic relationship of the isolated strain, enterobacterial repetitive intergenic consensus polymerase chain reaction (ERIC-PCR) was used to compare the isolated strain with control strains of *V. cholerae* O1 (Inaba and Ogawa/Classical) and *V. cholerae* O139 (Figure 5). All strains were fingerprinted using the following ERIC primers, as described elsewhere: ERIC1R (ATG TAA GCTCCT GGG GAT TCA C) and ERIC2 (AAGTAAGTGACTGGGGTGAGCG) [22]. The analysis of the ERIC-PCR fingerprint results using UPGMA and the cosine coefficient revealed that *V. cholerae* non-O1/O139 isolated from the wound infection grouped with the control strains of *V. cholerae* shared 94% genetic similarity (Figure 5).

## 3. Discussion

*Vibrio cholerae* is responsible for causing cholera, a very contagious diarrheal illness that impacts millions of people globally on annual basis [28,29]. Cholera poses a significant public health challenge, especially in nations with inadequate sanitation and areas prone to natural disasters, where the availability of clean drinking water is scarce [28]. Until now, only *V. cholerae* strains from serogroups O1 and O139 have been responsible for epidemic and pandemic cholera outbreaks, whereas strains from serogroups non-O1/non-O139 have been linked to sporadic cases of diarrhea and extraintestinal infections. Serogroups O1 and O139 exhibit pathogenicity through the production of cholera toxin (CT), which is carried in the genome of a filamentous bacteriophage known as CTXϕ [1]. Additionally, these serogroups are able to adhere to the intestine by utilizing toxin-coregulated pilus (TCP) as a colonization factor, which is encoded by a pathogenicity island. Non-O1/non-O139 *V. cholerae* strains are present in estuarine and coastal waters, yet their medical importance is often overlooked [29]. These NOVC strains have been linked to conditions such as septicemia, peritonitis, and gastroenteritis, typically through the consumption of contaminated food or contact with the aquatic surroundings [10,29,30]. Various potential virulence factors have been identified in NOVC, such as hemolysin (*hly*A), ToxR regulon (*tox*R), outer membrane proteins (*omp*U), and zonula occludens toxin (*zot*) [8,31,32,33]. In some cases, *V. cholerae* non-O1/non-O139 strains have also shown the presence of cholera toxin (*ctx*A) and toxin-coregulated pilus-associated genes (*tcp*A) [33,34].

In this report, we present a case of *V. cholerae* non-O1/O139 infection in a patient with a medical history of type II diabetes, hypertension, hypothyroidism, hypertension, a previous stroke 5 years ago resulting in residual weakness in her left lower limb, end-stage renal disease (ESRD), for which she was undergoing hemodialysis, recently diagnosed heart failure, hypothyroidism, and COVID-19 pneumonia. To the best of our knowledge, this is the first report to isolate toxigenic *V. cholerae* non-O1/O139 from a wound infection in a hospitalized patient in Saudi Arabia. Over 200 serogroups have been identified so far by examining the surface-expressed O antigen in *V. cholerae* strains. The *V. cholerae* strains that do not express the O1 and O139 antigens are commonly categorized as NOVC strains [1,3]. The NOVC strains have been linked to cases of moderate to severe gastroenteritis and extraintestinal infections like wound and soft tissue infections, ear infections, and bacteremia [9,11,12,13,20]. Initially, many of the NOVC strains were classified as nontoxigenic because they did not possess toxigenic CTX- and TCP-encoding genes [34]. However, in recent years, there has been an increase in cases of extraintestinal infections caused by *V. cholerae* non-O1/non-O139, with reports coming from various regions around the globe. These infections include septicemia, meningitis, cellulitis, and keratitis [11,28,35,36]. While *V. cholerae* is widely recognized as the agent responsible for cholera, i.e., a gastrointestinal illness acquired through contaminated food or water that results in severe dehydration due to profuse watery diarrhea, the non-O1/non-O139 strain can also cause cholera-like symptoms resembling those caused by the pandemic *V. cholerae* serogroups O1 and O139 [29]. Some NOVC strains may carry the cholera toxin gene or produce toxins similar to that found in many cases of diarrhea such as zot and ace, which are present with the same virulence cassette as the CT genes [37]. Unlike the non-invasive intestinal bacterial pathogens *V. cholerae* O1 and O139, which are primarily found in fecal stool specimens due to their inability to breach the intestinal mucosa, NOVC strains have the potential to invade tissues and cause septicemia and other infections outside of the intestinal tract [16,38].

At the beginning of 2020, the world witnessed the spread of SARS-CoV-2 as it spread into a global pandemic that led to over 40 million cases and over 2 million deaths worldwide by November 2020 [39]. In November of 2020, Saudi Arabia had over 300,000 cases with over 5000 deaths due to this virus [40]. However, the healthcare systems in various countries including Saudi Arabia have been greatly affected by the COVID-19 pandemic [41,42]. Several bacterial species, viruses, fungi, and parasites have been reported as associated coinfections with COVID-19 [43,44,45]. Among the reported Gram-negative and positive bacterial coinfections worldwide, this is the first case to report the isolation of toxigenic *V. cholerae* non-O1/O139 coinfection with a COVID-19 patient in Saudi Arabia. Based on this case study, it is crucial to report the identification of the isolated strain carrying the cholera toxin (CT).

Typically, NOVC strains do not possess the pathogenicity island of CT and TCP, but they do contain other virulence genes like zonula occludens toxin [46,47]. The NOVC strain in the current case was analyzed and compared with control strains of *V. cholerae* O1 serotype (Inaba and Ogawa) and the Bengal strain of *V. cholerae* O139 using ERIC-PCR to explore potential epidemiological connections. Consequently, the investigated NOVC strain was found to be closely related to the control strains, sharing 94% genetic similarity.

All recent reports from Saudia Arabia based on a PubMed search indicate that extraintestinal infections with NOVC may be more prevalent than *V. cholerae* O1 or O139 infections, which are considered rare [30,36,38,48,49]. This suggests that adding NOVC infections to the list of notifiable diseases may be warranted, especially after the pandemic, as it may have led to an increase in immunocompromised individuals, therefore making it easier for other strains to develop opportunistic characteristics or natures, therefore becoming coinfections easier than before. National surveillance for all Vibrio species would enhance our understanding of the impact and epidemiology of these potential pathogens and would provide valuable data for evaluating the effectiveness of interventions to manage Vibrio-related illnesses. Furthermore, monitoring the coastal environment is crucial, as NOVC can survive in seawater and on plankton during algal blooms, potentially leading to the contamination of local fish and seafood. In the current study, a limitation was the lack of a photograph showing the patient’s skin lesions.

## 4. Conclusions

*V. cholerae* non-O1/non-O139 infection may pose a significant risk and be life-threatening in patients with immunodeficiencies and can result in extraintestinal infections such as wound infections and primary septicemia. In light of this, it is important that, if a patient with a suspected NOVC infection develops a wound infection or an extraintestinal infection, antibiotic treatment and special care must be commenced promptly. This study marks the first instance of *V. cholerae* non-O1/non-O139 producing the cholera toxin being isolated from a patient with a bacteremic skin infection with multiple skin necrosis in Saudi Arabia.

## Figures and Tables

**Table 1:** Laboratory results.

Test	Result	Normal Range
CRP	26.71 mg/dL	0.1–0.5 mg/dL
Procalcitonin	4.46 μg/mL	0.063–0.7 μg/mL
WBC	17.8 k/μL	4.0–11.0 k/μL
Hgb	10.6 g/dL	12.0–16.0 g/dL
Plt	444 k/μL	140–450 k/μL
Random sugar	363 mg/dL	70–140 mg/dL
HbA1c	9.6%	4–6%
ESR	120 mm/h	0–20 mm/h

Abbreviations: CRP, C-reactive protein; WBC, white blood cell count; Hgb, hemoglobin level; Plt, platelet count; HbA1c, hemoglobin A1c; ESR, erythrocyte sedimentation rate.

**Table 2:** Sequences of primers used for detection of regulatory and selected virulence genes.

Target	Nucleotide Sequence (5′-3′)	Amplicon Size (bp)	Reference
*tox*R	CGGGATCCATGTTCGGATTAGGACAC CGGGATCCTACTCACACACTTTGATGGC	900	[23]
*omp*U	ACGCTGACGGAATCAACCA AAG GCGGAAGTTTGGCTTGAAG TAG	869	[24]
*ctx*A	CTCAGACGGGATTTGTTAGGCACG TCTATCTCTGTAGCCCCTATTACG	301	[25]
*zot*	TCGCTTAACGATGGCGCGTTTT AACCCCGTTTCACTTCTACCCA	947	[4]
*tcp*A	ACCAAATGCAACGCCGAATGGAGC GAAGAAGTTTGTAAAAGAAGAACAC	617	[26]
*ace*	TAAGGATGTGCTTATGATG GACACCC CGTGATGAATAAAGATACT CATAGG	316	[27]
*hly*A	GAGCCGGCATTCATCTGAAT CTCAGCGGGCTAATACGGTTTA	481	[4]

## Data Availability

The original contributions presented in the study are included in the article; further inquiries can be directed to the corresponding author.

## References

[1] Waldor M.K., Mekalanos J.J. (1996). Lysogenic Conversion by a Filamentous Phage Encoding Cholera Toxin. Science.

[2] Ramamurthy T., Mutreja A., Weill F.X., Das B., Ghosh A., Nair G.B. (2019). Revisiting the Global Epidemiology of Cholera in Conjuction with the Genomics of *Vibrio cholerae*. Front. Public Health.

[3] Yamai S., Okitsu T., Shimada T., Katsube Y. (1997). Distribution of Serogroups of *Vibrio cholerae* Non-O1 Non-O139 with Specific Reference to Their Ability to Produce Cholera Toxin, and Addition of Novel Serogroups. Kansenshogaku Zasshi..

[4] Singh D.V., Matte M.H., Matté G.R., Jiang S., Sabeena F., Shukla B.N., Sanyal S.C., Huq A., Colwell R. (2001). Molecular Analysis of *Vibrio cholerae* O1, O139, Non-O1, and Non-O139 Strains: Clonal Relationships between Clinical and Environmental Isolates. Appl. Environ. Microbiol..

[5] Zhang Q., Alter T., Fleischmann S. (2024). Non-O1/Non-O139 *Vibrio cholerae*—An Underestimated Foodborne Pathogen? An Overview of Its Virulence Genes and Regulatory Systems Involved in Pathogenesis. Microorganisms.

[6] Watve S., Barrasso K., Jung S.A., Davis K.J., Hawver L.A., Khataokar A., Palaganas R.G., Neiditch M.B., Perez L.J., Ng W.-L. (2019). Ethanolamine Regulates CqsR Quorum-Sensing Signaling in *Vibrio cholerae*. BioRxiv.

[7] Krysenko S., Wohlleben W. (2022). Polyamine and Ethanolamine Metabolism in Bacteria as an Important Component of Nitrogen Assimilation for Survival and Pathogenicity. Med. Sci..

[8] Ramamurthy T., Nandy R.K., Mukhopadhyay A.K., Dutta S., Mutreja A., Okamoto K., Miyoshi S.-I., Nair G.B., Ghosh A. (2020). Virulence Regulation and Innate Host Response in the Pathogenicity of *Vibrio cholerae*. Front. Cell. Infect. Microbiol..

[9] Pitrak D.L., Gindorf J.D. (1989). Bacteremic Cellulitis Caused by Non-Serogroup O1 *Vibrio cholerae* Acquired in a Freshwater Inland Lake. J. Clin. Microbiol..

[10] Dutta D., Chowdhury G., Pazhani G.P., Guin S., Dutta S., Ghosh S., Rajendran K., Nandy R.K., Mukhopadhyay A.K., Bhattacharya M.K. (2013). *Vibrio cholerae* Non-O1, Non-O139 Serogroups and Cholera-like Diarrhea, Kolkata, India. Emerg. Infect. Dis..

[11] Chowdhury G., Joshi S., Bhattacharya S., Sekar U., Birajdar B., Bhattacharyya A., Shinoda S., Ramamurthy T. (2016). Extraintestinal Infections Caused by Non-Toxigenic *Vibrio cholerae* Non-O1/Non-O139. Front. Microbiol..

[12] Rodríguez J.Y., Duarte C., Rodríguez G.J., Montaño L.A., Benítez-Peñuela M.A., Díaz P., López O., Álvarez-Moreno C.A. (2023). Bacteremia by Non-O1/Non-O139 *Vibrio cholerae*: Case Description and Literature Review. Biomedica.

[13] Aguinaga A., Portillo M.E., Yuste J.R., del Pozo J.L., García-Tutor E., Pérez-Gracia J.L., Leiva J. (2009). Non-O1 *Vibrio cholerae* Inguinal Skin and Soft Tissue Infection with Bullous Skin Lesions in a Patient with a Penis Squamous Cell Carcinoma. Ann. Clin. Microbiol. Antimicrob..

[14] Van Bonn S.M., Schraven S.P., Schuldt T., Heimesaat M.M., Mlynski R., Warnke P.C. (2020). Chronic Otitis Media Following Infection by Non-01/Non-0139 *Vibrio cholerae*: A Case Report and Review of the Literature. Eur. J. Microbiol. Immunol..

[15] Froelich B.A., Daines D.A. (2020). In Hot Water: Effects of Climate Change on Vibrio–Human Interactions. Environ. Microbiol..

[16] Deshayes S., Daurel C., Cattoir V., Parienti J.J., Quilici M.L., de La Blanchardière A. (2015). Non-O1, Non-O139 *Vibrio cholerae* Bacteraemia: Case Report and Literature Review. Springerplus.

[17] Zhang W., Xiao L., Shan X., Dai B., Tang C., Xian J., Yu Y. (2024). Case Report: Detection of Non-O1/Non-O139 *Vibrio cholerae* in a Patient with Hepatic Space-Occupying Lesions Using Metagenomic Next-Generation Sequencing. Front. Med..

[18] Vithiya G., Velvizhi S., Sundaram P.S., Mandal J. (2024). Non O1 Non O139 *Vibrio cholerae* Septicemia in a Patient with Carcinoma Pancreas-a Case Report and Literature Review, 2020–2023. Indian J. Med. Microbiol..

[19] Alex V., Moodley M. (2025). *Vibrio cholerae* Bacteraemia: A Case Report on an Unusual Presentation. Diagn. Microbiol. Infect. Dis..

[20] Marino A., Cacopardo B., Villa L., D’Emilio A., Piro S., Nunnari G. (2024). Think Vibrio, Think Rare: Non-O1-Non-O139-*Vibrio cholerae* Bacteremia in Advanced Lung Cancer—A Case Report. Trop. Med. Infect. Dis..

[21] Zhang X., Lu Y., Qian H., Liu G., Mei Y., Jin F., Xia W., Ni F. (2020). Non-O1, Non-O139 *Vibrio cholerae* (NOVC) Bacteremia: Case Report and Literature Review, 2015–2019. Infect. Drug Resist..

[22] Versalovic J., Koeuth T., Lupski R. (1991). Distribution of Repetitive DNA Sequences in Eubacteria and Application to Finerpriting of Bacterial Enomes. Nucleic Acids Res..

[23] Miller V.L., Taylor R.K., Mekalanos J.J. (1987). Cholera Toxin Transcriptional Activator ToxR Is a Transmembrane DNA Binding Protein. Cell.

[24] Rivera I.N.G., Chun J., Huq A., Sack R.B., Colwell R.R. (2001). Genotypes Associated with Virulence in Environmental Isolates of *Vibrio cholerae*. Appl. Environ. Microbiol..

[25] Shirai H., Nishibuchi M., Ramamurthy T., Bhattacharya S.K., Pal S.C., Takeda Y. (1991). Polymerase Chain Reaction for Detection of the Cholera Enterotoxin Operon of *Vibrio cholerae*. J. Clin. Microbiol..

[26] Keasler S., Hall R. (1993). Detecting and Biotyping *Vibrio cholerae* O1 with Multiplex Polymerase Chain Reaction. Lancet.

[27] Singh D.V., Isac S.R., Colwell R. (2002). Development of a Hexaplex PCR Assay for Rapid Detection of Virulence and Regulatory Genes in *Vibrio cholerae* and *Vibrio mimicus*. J. Clin. Microbiol..

[28] Montero D.A., Vidal R.M., Velasco J., George S., Lucero Y., Gómez L.A., Carreño L.J., García-Betancourt R., O’Ryan M. (2023). *Vibrio cholerae*, Classification, Pathogenesis, Immune Response, and Trends in Vaccine Development. Front. Med..

[29] Baker-Austin C., Oliver J.D., Alam M., Ali A., Waldor M.K., Qadri F., Martinez-Urtaza J. (2018). *Vibrio* spp. Infections. Nat. Rev. Dis. Prim..

[30] Issa H. (2009). A Case of O1 Vibrio Cholera Bacteremia and Primary Peritonitis in a Patient with Liver Cirrhosis. Gastroenterol. Res..

[31] Schmidt K., Scholz H.C., Appelt S., Michel J., Jacob D., Dupke S. (2023). Virulence and Resistance Patterns of *Vibrio cholerae* Non-O1/Non-O139 Acquired in Germany and Other European Countries. Front. Microbiol..

[32] Saravanan V., Sanath Kumar H., Karunasagar I., Karunasagar I. (2007). Putative Virulence Genes of *Vibrio cholerae* from Seafoods and the Coastal Environment of Southwest India. Int. J. Food Microbiol..

[33] Hasan N.A., Ceccarelli D., Grim C.J., Taviani E., Choi J., Sadique A., Alam M.C., Siddique A.K., Bradley Sack R., Huq A. (2013). Distribution of Virulence Genes in Clinical and Environmental *Vibrio cholerae* Strains in Bangladesh. Appl. Environ. Microbiol..

[34] Bhandari M., Rathnayake I.U., Huygens F., Nguyen S., Heron B., Jennison A.V. (2023). Genomic and Evolutionary Insights into Australian Toxigenic *Vibrio cholerae* O1 Strains. Microbiol. Spectr..

[35] Chen W.D., Lai L.J., Hsu W.H., Huang T.Y. (2019). *Vibrio cholerae* Non-O1—The First Reported Case of Keratitis in a Healthy Patient. BMC Infect. Dis..

[36] Abdelhafiz T.A., Alnimr A.M., Alabduljabbar A.M., AlMuqallad H.S., Abdulmonem Alzarra A., Alrashed H.N., Aladwani M.M., Hakami A.M. (2019). Non O1 *Vibrio cholerae* as a Cause of Bacteremic Lower Limb Cellulitis: A Case Report. Int. J. Surg. Case Rep..

[37] Takahashi E., Ochi S., Mizuno T., Morita D., Morita M., Ohnishi M., Koley H., Dutta M., Chowdhury G., Mukhopadhyay A.K. (2021). Virulence of Cholera Toxin Gene-Positive *Vibrio cholerae* Non-O1/Non-O139 Strains Isolated from Environmental Water in Kolkata, India. Front. Microbiol..

[38] Aljindan R., Allahham R., Alghamdi R., Alhabib I., Alnassri S., Alkhalifa W., Diab A., Alomar A., Yamani L., Elhadi N. (2024). Isolation and Characterization of Cholera Toxin Gene-Positive *Vibrio cholerae* Non-O1/Non-O139 Isolated from Urinary Tract Infection: A Case Report. Infect. Drug Resist..

[39] Msemburi W., Karlinsky A., Knutson V., Aleshin-Guendel S., Chatterji S., Wakefield J. (2023). The WHO Estimates of Excess Mortality Associated with the COVID-19 Pandemic. Nature.

[40] Alswaidi F.M., Assiri A.M., Alhaqbani H.H., Alalawi M.M. (2021). Characteristics and Outcome of COVID-19 Cases in Saudi Arabia: Review of Six-Months of Data (March–August 2020). Saudi Pharm. J..

[41] Alenzi K.A., Al-malky H.S., Altebainawi A.F., Abushomi H.Q., Alatawi F.O., Atwadi M.H., Khobrani M.A., Almazrou D.A., Alrubeh N., Alsoliabi Z.A. (2022). Health Economic Burden of COVID-19 in Saudi Arabia. Front. Public Health.

[42] Arsenault C., Gage A., Kim M.K., Kapoor N.R., Akweongo P., Amponsah F., Aryal A., Asai D., Awoonor-Williams J.K., Ayele W. (2022). COVID-19 and Resilience of Healthcare Systems in Ten Countries. Nat. Med..

[43] Dueñas D., Daza J., Liscano Y. (2023). Coinfections and Superinfections Associated with COVID-19 in Colombia: A Narrative Review. Medicina.

[44] Scott H., Zahra A., Fernandes R., Fries B.C., Thode Jr H.C., Singer A.J. (2022). Bacterial Infections and Death among Patients with COVID-19 versus Non COVID-19 Patients with Pneumonia. Am. J. Emerg. Med..

[45] Alqahtani A., Alamer E., Mir M., Alasmari A., Alshahrani M.M., Asiri M., Ahmad I., Alhazmi A., Algaissi A. (2022). Bacterial Coinfections Increase Mortality of Severely Ill COVID-19 Patients in Saudi Arabia. Int. J. Environ. Res. Public Health.

[46] Pérez-Reytor D., Jaña V., Pavez L., Navarrete P., García K. (2018). Accessory Toxins of *Vibrio* Pathogens and Their Role in Epithelial Disruption during Infection. Front. Microbiol..

[47] Zhao Y., He T., Tu B., Mao X., Jiang J., Jiang X., Wang F., Wang M., Wang Y., Sun H. (2022). Death in a Farmer with Underlying Diseases Carrying *Vibrio cholerae* Non-O1/Non-O139 Producing Zonula Occludens Toxin. Int. J. Infect. Dis..

[48] Nagamani R., Al Momen H.A.M. (2017). Non O1 *Vibrio Cholerae*: An Emerging Pathogen in Blood?—A Review and Report of Cases from a Regional Laboratory at the Eastern Province in Saudi Arabia. MRIMS J. Health Sci..

[49] Kaki R., El-Hossary D., Jiman-Fatani A., Al-Ghamdi R. (2017). Non-O1/Non-O139 *Vibrio cholerae* Septicaemia in a Saudi Man: A Case Report. JMM Case Rep..

